# Integrating AI in Healthcare Education: Attitudes of Pharmacy Students at King Khalid University Towards Using ChatGPT in Clinical Decision-Making

**DOI:** 10.3390/healthcare13111265

**Published:** 2025-05-27

**Authors:** Rajalakshimi Vasudevan, Taha Alqahtani, Saud Alqahtani, Praveen Devanandan, Geetha Kandasamy, Reema Saad, Asayel Amer, Raghad Abduallah, Ghada Waleed, Rahaf Hasan, Lamis Ahmed

**Affiliations:** 1 Department of Pharmacology, College of Pharmacy, King Khalid University, Abha 62521, Saudi Arabia; ttaha@kku.edu.sa (T.A.); saalqhtany@kku.edu.sa (S.A.); zaydryma@gmail.com (R.S.); asayel0@outlook.sa (A.A.); raghadabduallah@gmail.com (R.A.); ghadawaleed99@gmail.com (G.W.); rahafsan@gmail.com (R.H.); lamisahmed@gmail.com (L.A.); 2 Department of Pharmacy Practice, St. Peter’s Institute of Pharmaceutical Sciences, Hanamkonda 506001, Telangana, India; praveendevanandan@gmail.com; 3 Department of Clinical Pharmacy, College of Pharmacy, King Khalid University, Abha 62521, Saudi Arabia; glakshmi@kku.edu.sa

**Keywords:** attitude, practices, artificial intelligence, pharmacy students, ChatGpt, clinical decision-making

## Abstract

**Background:** Artificial Intelligence (AI) is transforming healthcare education, offering innovative tools to enhance learning and clinical decision-making. AI-driven platforms like ChatGPT are increasingly integrated into pharmacy education, enabling students to access vast medical knowledge, refine decision-making skills, and simulate clinical scenarios. **Objective:** This study examines pharmacy students’ attitudes, knowledge, and practices regarding ChatGPT’s use in clinical decision-making, evaluates its perceived benefits and limitations, and identifies factors influencing AI integration in pharmacy education. **Methodology**: A cross-sectional study was conducted among 512 pharmacy students at King Khalid University. A structured questionnaire assessed demographics, knowledge, attitudes, and practices. Data were analyzed using SPSS, employing descriptive statistics, chi-square tests, and logistic regression. **Results:** The majority (82.4%) supported AI integration in pharmacy education, while 74.6% believed that ChatGPT could enhance clinical decision-making. Primary applications included drug information retrieval (72.3%) and exam preparation (66.7%). However, concerns about AI accuracy (55.2%) and ethical implications were noted. **Conclusions:** Pharmacy students at King Khalid University exhibit positive attitudes toward AI, recognizing its educational benefits while acknowledging challenges. Addressing accuracy concerns and ethical considerations through structured AI training programs is essential to optimize AI’s role in pharmacy education and practice.

## 1. Introduction

Integrating Artificial Intelligence (AI) into healthcare and education has revolutionized traditional learning paradigms, offering innovative tools to enhance knowledge acquisition and application. In pharmacy education, AI-driven platforms such as ChatGPT-4O are becoming indispensable for developing future healthcare professionals. While generative AI tools offer innovative opportunities in pharmacy education, their integration remains a subject of active academic and ethical debate, particularly regarding reliability, equity, and their impact on critical thinking. These chat-based systems simulate human-like interactions, enabling students to engage with vast repositories of medical knowledge dynamically and personally. This aligns with the increasing demands of precision medicine and data-driven healthcare, where pharmacists must make critical decisions based on complex datasets [1,2].

AI tools like ChatGPT have shown tremendous potential in bridging the gap between theoretical learning and clinical practice. These tools empower pharmacy students to understand intricate pharmaceutical concepts, develop clinical reasoning, and enhance decision-making capabilities by providing instant responses, real-time feedback, and evidence-based insights. A study conducted at Afe Babalola University, Nigeria, demonstrated that over 80% of pharmacy students actively use ChatGPT to study, complete assignments, and access drug information. This high adoption rate reflects the growing recognition of AI’s value in supplementing traditional educational resources [3]. Additionally, studies across regions such as Jordan, Saudi Arabia, and Palestine have reported similar findings, highlighting widespread awareness and positive perceptions of AI tools among pharmacy students, further cementing their relevance in contemporary healthcare education [4,5,6].

One of the critical benefits of AI in pharmacy education lies in its ability to provide personalized learning experiences. While experienced educators often tailor content to individual needs, AI tools like ChatGPT offer scalable, 24/7 personalization that complements traditional instruction, particularly in large cohorts or outside regular classroom hours. This flexibility is particularly beneficial for pharmacy students, who often face demanding schedules [7,8].

Beyond academic learning, ChatGPT plays a significant role in preparing students for real-world clinical challenges. The tool’s ability to simulate clinical scenarios, analyze patient data, and provide treatment recommendations equips students with the skills needed for evidence-based decision-making. These capabilities are invaluable in an era where pharmacists are integral to multidisciplinary healthcare teams. For example, ChatGPT can assist students in identifying potential drug interactions, optimizing dosage regimens, and evaluating treatment outcomes, all of which are crucial for personalized patient care [9,10].

Despite these advantages, integrating AI tools into pharmacy education is challenging. Concerns about the accuracy and reliability of AI-generated information remain prevalent. While ChatGPT can provide rapid responses, its reliance on pre-existing datasets raises the risk of propagating outdated or incorrect information. This issue underscores the importance of training students to critically evaluate AI outputs rather than accepting them at face value. Furthermore, ethical concerns such as academic dishonesty and over-reliance on AI tools have emerged. A study from Nigeria revealed that 65% of students believed that using AI tools like ChatGPT could increase the likelihood of academic misconduct. This highlights the need for clear guidelines and regulations to ensure the responsible use of AI in educational settings [3,11,12,13].

Another significant barrier to AI integration is the lack of standardized curricula incorporating these tools. While some institutions have embraced AI as a core component of pharmacy education, many institutions have yet to adopt AI tools, which may reflect legitimate concerns regarding integration strategies, training, ethical implications, or resource constraints. This disparity creates unequal learning opportunities for students and underscores the need for policy interventions to promote the widespread adoption of AI in pharmacy programs. Faculty training is equally critical to address misconceptions about AI replacing traditional teaching methods. Studies have shown that effective faculty involvement can enhance integration, ensuring that AI tools complement rather than substitute human educators [4,7,14]

In clinical decision-making, ChatGPT’s potential extends beyond student learning to improving patient outcomes. The tool can analyze patient-specific factors, such as genetic profiles and lifestyle data, to recommend personalized treatments. This aligns with the principles of precision medicine and highlights the evolving role of AI in shaping modern healthcare practices. However, these applications also demand robust ethical frameworks to address data privacy and algorithmic bias. Educators and policymakers must work together to create an environment where AI tools are used ethically and effectively [10,15].

Recognizing these gaps, this study explores several key aspects of AI integration in pharmacy education. The objectives are fourfold: [i] to assess the attitudes of pharmacy students towards using ChatGPT for clinical decision-making, [ii] to evaluate the practices of pharmacy students in incorporating ChatGPT into their educational and clinical decision-making processes, [iii] to analyze their knowledge regarding the benefits and limitations of AI in pharmacy practice, and [iv] to identify factors influencing the integration of AI tools in pharmacy education. Through these objectives, we aim to comprehensively understand how pharmacy students perceive, use, and benefit from AI tools like ChatGPT while identifying challenges that may hinder their effective utilization. Furthermore, understanding these dimensions will help educators and policymakers design curricula that align with technological advancements, ultimately equipping future pharmacists with the skills needed to excel in an increasingly digital healthcare landscape.

## 2. Methodology

### 2.1. Study Design

This research adopted a cross-sectional design to evaluate pharmacy students’ knowledge, attitudes, and practices (KAP) regarding ChatGPT’s use in clinical decision-making.

### 2.2. Study Setting and Participants

A stratified convenience sampling approach was used to proportionally include pharmacy students from all academic years (first to fourth year), as well as interns, at King Khalid University. Recruitment took place through email invitations and social media. Participants included pharmacy students who had direct or indirect exposure to ChatGPT or AI tools.

### 2.3. Sample Size Calculation

The sample size (*n* = 512) was calculated using a population proportion formula at a 95% confidence interval, a 5% margin of error, and a prevalence estimate of 50% for AI usage.

### 2.4. Data Collection Tool: Questionnaire

A structured questionnaire was designed, comprising four sections: demographics, knowledge, attitudes, and practices. This tool was adapted from validated instruments used in similar studies [16,17,18,19] and refined to align with the study’s objectives. Expert AI and pharmacy education reviewers assessed the questionnaire for content validity, ensuring its relevance and clarity. The demographics section collected data on age, gender, academic year, and prior AI experience. Knowledge items (five questions) evaluated understanding of AI benefits and limitations, while attitude items (five questions) assessed perceptions of ChatGPT’s reliability and accuracy. Practice items (five questions) explored the frequency and purpose of ChatGPT use in educational and clinical contexts.

A pilot test involving 30 participants assessed reliability, yielding a Cronbach’s alpha score of 0.69, consistent with reliability thresholds reported by Huang et al. [16]. Feedback from the pilot test informed minor revisions for clarity and usability.

### 2.5. Data Collection Procedure

Data collection was conducted online using Google Forms. Participants were provided with a link to the survey and an information sheet explaining the study’s purpose, voluntary nature, and data confidentiality. Recruitment was conducted via the university email system, student Telegram and WhatsApp groups, and through class representatives. Weekly reminders were sent over a 4-week period to enhance response rates, which Patel et al. [14] and Amin et al. [15] also utilized.

The survey took approximately 10–15 min, and responses were automatically recorded on a secure platform. Participants provided informed consent electronically before starting the study.

### 2.6. Ethical Considerations

Ethical approval was obtained from the Research Ethics Committee at King Khalid University HAPO-06-B-001 with the approval number ECM#2024-3130. Participants’ anonymity and confidentiality were safeguarded, and data were stored on encrypted servers.

### 2.7. Data Analysis

Statistical analysis was performed using SPSS software Version 21.0. Descriptive statistics, such as frequencies and percentages, were used to summarize the demographic characteristics and Likert-scale responses. Inferential statistics included chi-square tests to identify associations between demographic factors and KAP responses and logistic regression to determine positive attitudes and frequent ChatGPT usage predictors.

## 3. Results

### 3.1. Participant Demographics

Out of approximately 800 students invited, 512 responded, yielding a response rate of 64%. A total of 512 pharmacy students participated; their demographic details are shown in Table 1.

A total of 512 pharmacy students were involved in the study, of which 60% were female (*n* = 307) and 40% were male (*n* = 205). The average age of participants was 22.0 ± 2.1 years. The students were proportionately allocated among their academic years: first year (19.9%), second year (27.3%), third year (24.8%), and fourth year (27.9%). Notably, 77.7% of participants indicated prior knowledge of AI technologies. Although 22.3% of participants reported no prior direct use, their responses were included to capture general attitudes and perceived barriers.

### 3.2. Attitudes Toward AI Tools

The survey indicated predominantly favorable perceptions of AI tools such as ChatGPT. Approximately 82.4% of individuals concurred that AI tools should be incorporated into pharmacy instruction, indicating substantial excitement for embracing technology breakthroughs in learning.

Approximately 74.6% believed that ChatGPT might improve clinical decision-making abilities (*p* < 0.01), whereas 68.1% expressed ease in using the technology for clinical inquiries (*p* < 0.05). Nonetheless, 55.2% voiced apprehensions regarding the accuracy of ChatGPT, but this issue did not attain statistical significance (*p* = 0.081).

Participants’ sentiments toward ChatGPT were summarized, and chi-square tests were utilized to evaluate correlations with prior experience and year of study (Table 2).

### 3.3. Practices and Applications

ChatGPT was extensively utilized among the participants for various educational and practical applications. The predominant application was medication information retrieval (72.3%, mean Likert score: 4.2 ± 0.8), followed by preparation for examinations and assignments (66.7%, mean Likert score: 4.0 ± 0.7). Additional uses encompassed the examination of clinical guidelines (53.4%), facilitation of patient counseling situations (48.6%), and execution of research reviews (40.9%). Usage trends were examined, concentrating on ChatGPT’s principal applications. The predominant application was retrieving drug information (Table 3).

### 3.4. Knowledge of AI Tools

Participants demonstrated a robust understanding of AI’s advantages, with 86.1% acknowledging its capacity to enhance healthcare procedures (*p* < 0.01). A significant 67.4% concurred that AI could improve patient care (*p* < 0.05), while 43.8% voiced apprehensions regarding the dangers of excessive dependence on AI systems. Participants exhibited differing degrees of comprehension regarding the advantages and constraints of AI tools (Table 4).

### 3.5. Perceived Impact on Education and Practice

The perceived influence of ChatGPT on education and practice was examined via logistic regression to determine predictors of favorable views (Table 5). A significant majority of respondents (85.7%) asserted that AI tools enhanced personalized learning experiences (OR: 2.1, CI: 1.6–2.8, *p* < 0.01). In a similar vein, 81.2% endorsed the incorporation of AI into pharmacy education (OR: 1.8, CI: 1.3–2.4, *p* < 0.05), while 71.6% believed that AI may improve pharmacy practice results (OR: 1.5, CI: 1.1–2.0, *p* = 0.048).

### 3.6. Challenges in Using ChatGPT

Participants identified obstacles to the effective use of ChatGPT. The challenges were systematically categorized and analyzed (see Table 6). Participants recognized various challenges related to the utilization of ChatGPT. The predominant challenge identified was verifying the accuracy of AI-generated information, reported by 51.7% of respondents (*p* < 0.01). Additional notable challenges comprised insufficient training in effective AI utilization (43.2%, *p* < 0.05) and ethical apprehensions regarding over-reliance (36.4%, *p* = 0.03).

## 4. Discussion

This study evaluated pharmacy students’ demographics, attitudes, practices, and knowledge concerning using ChatGPT in clinical decision-making. The findings reveal an overall positive perception of AI, specifically ChatGPT, as an educational and clinical support tool. Yet, they also underscore a balanced approach among students, who were mindful of potential risks and limitations. The findings of this study demonstrate the increasing acceptance and utility of AI tools like ChatGPT among pharmacy students. These findings are consistent with those from Xiong et al. (2023) [20] and Lin et al. (2023) [21], where students in China and Singapore similarly reported a strong acceptance of AI in education. Similarly, Mosleh et al. (2023) [4] reported positive AI perceptions among students in Jordan and Palestine, reinforcing the global trend. These results align with global trends emphasizing the integration of AI into healthcare education to prepare future professionals for a rapidly evolving technological landscape. The findings align with constructivist learning theory, which emphasizes learner engagement and active knowledge construction, a process facilitated by ChatGPT’s interactive features. Additionally, the ability to tailor queries and receive scaffolded responses reflects higher-order thinking skills consistent with Bloom’s taxonomy, particularly at the ‘application’ and ‘analysis’ levels [22].

The positive attitudes toward AI integration reflect a shift in educational expectations. Similar studies have noted that pharmacy and healthcare students perceive AI as a critical tool for bridging knowledge gaps and enhancing decision-making skills [21]. This study further reinforces that students recognize the potential for tools like ChatGPT to complement traditional learning methods by providing quick, evidence-based insights into complex topics. ChatGPT’s primary use for drug information retrieval (72.3%) highlights its utility in addressing one of pharmacy practice’s most time-sensitive tasks. This finding aligns with research showing that AI-driven tools reduce workload and improve accuracy in pharmacy operations [23]. However, less frequent use in areas like literature reviews (40.9%) may indicate that students are less confident in applying AI for research-intensive tasks.

The high awareness of AI’s benefits (86.1%) contrasts with the lower awareness of ethical concerns (61.2%). This discrepancy underscores the need for structured AI literacy programs emphasizing AI’s advantages and limitations in healthcare settings. Studies suggest that integrating AI ethics into curricula improves students’ ability to assess AI recommendations critically and fosters responsible use [23].

A notable finding in our study is the generally optimistic view among students about ChatGPT’s role in enhancing clinical decision-making skills, with nearly 70% expressing confidence in its potential. This mirrors the findings of a recent study by Xiong et al. [20], which explored medical and pharmacy students’ attitudes toward AI in clinical contexts. About 72% of participants in the survey agreed that AI could benefit clinical decision-making. Both studies indicate a shared optimism regarding AI’s educational benefits. However, our cohort’s 30% neutral or negative responses highlight a higher level of caution than that observed in Xiong et al.’s study. This discrepancy could be attributed to different levels of exposure or experience with AI, as our cohort showed a wide range of academic levels, from first-year students to interns and practitioners.

The positive comfort levels observed in our study, with around 69% feeling at ease using ChatGPT for clinical queries, are also consistent with Xiong et al.’s findings. In both studies, comfort and acceptance of AI were influenced by familiarity with digital tools and a growing expectation that AI would be integrated into healthcare education. However, the concern for accuracy in our study—where 76% were cautious about ChatGPT’s reliability—echoes the mixed sentiments from another study by Patel et al. (2023) [14], which found that about 68% of healthcare students expressed similar concerns. This reinforces a critical point: while students value AI tools, they recognize that tools like ChatGPT must be used discerningly to avoid over-reliance, especially in critical clinical situations.

### 4.1. Practices of Using ChatGPT in Academic and Clinical Contexts

Our results also illustrate varied levels of ChatGPT use in different practical contexts. For instance, around 53% of students in our study used ChatGPT for drug information retrieval. This aligns with findings from a recent survey by Lin et al. (2023) [21], where 55% of pharmacy and medical students reported using AI to supplement drug information searches. This similarity suggests a consistent perception of AI as a quick-reference tool. However, our study showed a more reserved use of ChatGPT in exam preparation, with nearly 46% disagreeing. This contrasts with Lin et al., where almost 60% of students reported using AI tools, including ChatGPT, for study purposes. The difference may be due to varying confidence levels in ChatGPT’s accuracy for test preparation, underscoring a desire for more trustworthy and vetted information in exam contexts.

Our findings regarding ChatGPT’s use in patient counseling scenarios, where nearly 49% of students found it helpful, also correspond with the moderate engagement levels seen in recent studies. The study by Amin et al. [15] similarly noted a cautious acceptance among students for using AI in simulated patient scenarios, highlighting the perceived advantages in helping structure responses and the limitations of potential inaccuracies in real-time counseling. Thus, the balanced usage patterns seen in our study support the trend observed across recent research: students see ChatGPT as a helpful tool in clinical learning but approach direct patient-related scenarios with prudence, possibly due to ethical considerations and the potential for misinformation.

### 4.2. Knowledge of AI Benefits and Limitations

Our study’s findings on the knowledge of AI benefits and limitations underscore a well-rounded understanding among pharmacy students, with about 78% recognizing AI’s potential benefits and a similar percentage acknowledging limitations and ethical considerations. This awareness aligns closely with the findings of Xu et al. [23], who reported that approximately 80% of healthcare students acknowledged both the potential and limitations of AI tools in practice. This similarity across studies highlights a widespread awareness of AI’s dual role as both a powerful tool and a resource requiring responsible use.

Additionally, the substantial concern expressed by students in our study regarding the risks of over-reliance on AI (with around 78% in agreement) reflects findings in other recent studies. For example, Huang et al. [16] found that nearly 75% of pharmacy students raised concerns about becoming overly dependent on AI tools, citing a risk to clinical judgment and critical thinking skills. Concerns about accuracy and over-reliance highlight a critical need for educational strategies that promote AI literacy. These include teaching students to triangulate AI responses with evidence-based resources and develop critical thinking skills, preventing blind trust in automated outputs. Such concerns are echoed in studies by Huang et al. (2023) [16], who found that over 70% of students feared diminished clinical reasoning due to AI dependence. Both studies indicate a growing understanding that while AI can enhance efficiency and learning, it should complement, rather than replace, traditional, evidence-based clinical education and practice.

Participants’ concerns about verifying AI-generated information and ethical issues reflect broader challenges in AI adoption. While AI tools like ChatGPT are powerful, their reliance on probabilistic outputs makes them prone to inaccuracies. Addressing this requires developing robust training programs that teach students to cross-verify AI outputs against established clinical guidelines.

Our findings are generally consistent with other recent studies, such as those by Xiong et al., Lin et al., and Amin et al., indicating shared attitudes among healthcare students regarding AI’s educational and clinical utility. However, our study’s slightly higher levels of caution and neutrality could be attributed to our participants’ varying prior exposure to AI tools and potential differences in curriculum emphasis on AI across institutions. Additionally, the balanced views on ethical concerns and accuracy noted in our study are mirrored in the works of Patel et al. and Xu et al. [14,23], emphasizing a need for transparency in AI applications and critical assessment in clinical environments.

These results strongly support integrating AI tools into pharmacy education, with 81.2% of participants advocating for such inclusion. This finding echoes recommendations in the recent literature, where authors emphasize the role of AI in fostering adaptive learning environments and improving critical thinking skills [24]. However, successful integration requires a balanced approach that ensures students understand AI use’s limitations and ethical implications.

This study is limited by its cross-sectional design and focus on a single institution, which may restrict the generalizability of its findings. One limitation of this study is the moderate survey response rate of approximately 64%. While this response rate is acceptable for online surveys, there is a potential for non-response bias. Students who were more familiar with or interested in AI tools like ChatGPT may have been more likely to participate, possibly skewing the results toward more favorable attitudes and usage patterns. Therefore, the findings may not fully represent the views of the entire student population. Additionally, self-reported data may introduce bias, as participants could overstate their familiarity or comfort with AI tools. Future research should adopt longitudinal designs and include a diverse population to capture broader AI acceptance and use trends among pharmacy students.

### 4.3. Implications for Education and Practice

These findings collectively suggest that while pharmacy students are open to adopting AI in their education, they seek structured guidance and clear ethical frameworks for its use. The high support for AI training in pharmacy curricula indicates a demand for more comprehensive, formal instruction in AI applications. Future studies could explore the specific content and structure of AI curricula that would best prepare students to integrate these tools responsibly in practice.

## 5. Conclusions

This study highlights the positive attitudes of pharmacy students at King Khalid University toward AI tools and their potential to transform education and practice. While students recognize the benefits of tools like ChatGPT, challenges such as accuracy verification and ethical concerns need to be addressed.

Also, in the future, pharmacy programs should incorporate AI training modules focusing on practical applications, ethics, and critical evaluation skills. Future studies should explore the long-term outcomes of AI integration on academic performance and professional competency. Policy Implementation: Institutions should establish guidelines for the ethical use of AI in education and clinical practice. Pharmacy education can leverage AI to produce more competent and adaptable healthcare professionals by addressing these areas.

## Figures and Tables

**Table 1:** Participant demographics.

Variable	Frequency (*n* = 512)	Percentage (%)
Male	205	40.0
Female	307	60.0
Age (Mean ± SD)	22.0 ± 2.1 years	-
Year of Study		
- First Year	102	19.9
- Second Year	140	27.3
- Third Year	127	24.8
- Fourth Year	143	27.9
Prior AI Experience	398	77.7
No AI Experience	114	22.3

**Table 2:** Attitudes toward ChatGPT.

Question	Agreement (%)	*p*-Value (Chi-Square)
ChatGPT improves clinical decision-making skills	74.62	<0.01 *
Comfortable using ChatGPT for clinical queries	68.17	<0.05 *
ChatGPT provides reliable pharmacy information	65.72	<0.05 *
Concerned about the accuracy of ChatGPT	55.26	0.08123
AI tools like ChatGPT should be integrated	82.44	<0.01 *

* *p* < 0.05, considered statistically significant.

**Table 3:** Practices and applications of ChatGPT.

Practice Area	Usage (%)	Mean ± SD (Likert Scale: 1–5)	*p*-Value (Chi-Square)
Searching for drug information	72.31	4.2 ± 0.8	<0.01 *
Assisting with patient counseling scenarios	48.66	3.6 ± 0.9	0.02 *
Preparing for exams or assignments	66.73	4.0 ± 0.7	<0.05 *
Exploring clinical guidelines or protocols	53.42	3.8 ± 0.8	<0.05 *
Conducting literature reviews or research	40.90	3.2 ± 1.0	0.06112

* *p* < 0.05, considered statistically significant.

**Table 4:** Knowledge about AI tools.

Question	Agreement (%)	*p*-Value (Chi-Square)
Understanding the potential benefits of AI in healthcare practice	86.14	<0.01 *
Awareness of limitations and ethical concerns	61.23	<0.05 *
A belief that AI can enhance patient care	67.45	<0.05 *
Concerned about the risks of over-reliance	43.86	0.08641

* *p* < 0.05, considered statistically significant.

**Table 5:** Perceived impact of ChatGPT.

Perception Item	Agreement (%)	Odds Ratio (OR)	Confidence Interval (CI)	*p*-Value
AI tools improve personalized learning	85.74	2.1	1.6–2.8	<0.01 *
AI should be part of pharmacy education training	81.24	1.8	1.3–2.4	<0.05 *
AI integration enhances pharmacy practice outcomes	71.62	1.5	1.1–2.0	0.048 *

* *p* < 0.05, considered statistically significant.

**Table 6:** Challenges in using ChatGPT.

Challenge	Frequency (%)	*p*-Value (Chi-Square)
Difficulty verifying accuracy of AI-provided data	51.76	<0.01 *
Lack of training on effective use of AI tools	43.24	<0.05 *
Ethical concerns about reliance on AI	36.42	0.03 *
Difficulty contextualizing AI recommendations	30.57	0.09451

* *p* < 0.05, considered statistically significant.

## Data Availability

The original contributions presented in this study are included in the article. Further inquiries can be directed to the corresponding author.
